# Global identification and analysis of isozyme-specific possible substrates crosslinked by transglutaminases using substrate peptides in mouse liver fibrosis

**DOI:** 10.1038/srep45049

**Published:** 2017-03-22

**Authors:** Hideki Tatsukawa, Yuji Tani, Risa Otsu, Haruka Nakagawa, Kiyotaka Hitomi

**Affiliations:** 1 Cellular Biochemistry Lab., Graduate School of Pharmaceutical Sciences, Nagoya University, Furo-cho, Chikusa, Nagoya 464-8601, Japan

## Abstract

The transglutaminase (TG) family comprises eight isozymes that form the isopeptide bonds between glutamine and lysine residues and contribute to the fibrotic diseases via crosslinking-mediated stabilization of ECM and the activation of TGF-β in several tissues. However, despite a growing body of evidence implicating TG2 as a key enzyme in fibrosis, the causative role of TG2 and the involvement of the other isozymes have not yet been fully elucidated. Therefore, here we clarified the distributions of TG isozymes and their *in situ* activities and identified the isozyme-specific possible substrates for both TG1 and TG2 using their substrate peptides in mouse fibrotic liver. We found that TG1 activity was markedly enhanced intracellularly over a widespread area, whereas TG2 activity increased in the extracellular space. In total, 43 and 42 possible substrates were identified for TG1 and TG2, respectively, as involved in chromatin organization and cellular component morphogenesis. These included keratin 18, a biomarker for hepatic injury, which was accumulated in the fibrotic liver and showed the partly similar distribution with TG1 activity. These findings suggest that TG1 activity may be involved in the functional modification of intracellular proteins, whereas TG2 activity contributes to the stabilization of extracellular proteins during liver fibrosis.

Transglutaminases (TGs) are crosslinking enzymes that catalyze the formation of covalent bonds between glutamine (Gln) and lysine (Lys) residues in substrate proteins via Ca^2+^-dependent posttranslational modifying reactions^1^,^2^,^3^,^4^,^5^. The TG family comprises eight isozymes designated as factor XIIIa (FXIIIa) and TG1–7, which are widely distributed and involved in multiple biological processes, including blood coagulation, epidermis formation, transcriptional regulation, and extracellular matrix stabilization. In addition to these physiological functions, these is strong evidence that TGs are involved in a number of pathologies, including liver and skin diseases, cancer, inflammation, and neurodegeneration.

Several previous studies have investigated the involvement of TGs in liver disease, which have shown that only TG2 appears to play a role. However, although TG2 expression and activity significantly increased in a number of *in vitro* and *in vivo* models for liver injury and was involved in the progression of hepatic apoptosis and liver fibrosis^6^,^7^,^8^,^9^,^10^, the opposite effects have also been reported, even in the same model^11^,^12^,^13^, likely owing to the different cell types and animal backgrounds used^5^. Therefore, further detailed studies on the distributions of TG expressions and activities as well as on the identification of disease-specific substrates for each TG isozyme are required.

We previously characterized the preferred Gln-donor substrate sequences with a unique reaction tendency for each TG isozyme, using a random peptide library^14^,^15^. These peptide sequences appeared to act as substrates with high reactivity and isozyme specificity, even in the peptide form (12 amino acid residues). In addition, we developed a method for detecting isozyme-specific activities by incorporating a fluorescence-labeled substrate peptide into the Lys residues of proteins^16^. This revealed that the expressions and activities of both TG1 and TG2 were markedly higher in the liver than in the other internal organs^16^. Previous studies have shown that TG1 is mainly involved in skin formation, contributing to the barrier function of the outermost layers via cooperative crosslinking of structural proteins in keratinocytes^3^, whereas TG2 is widely distributed and plays multiple roles, including apoptosis, several types of signal transduction, matrix stabilization, wound healing, and angiogenesis^17^.

To elucidate the detailed mechanism whereby which TG1 and TG2 contribute to liver fibrotic diseases, here we developed a system able comprehensively to identify the possible substrate proteins incorporated in each isozyme-specific substrate peptide for TG1 and TG2, as well as biotinylated pentylamine (BPA). This led to several possible substrates being identified, among which we chose to focus on keratin 8 (K8) and keratin 18 (K18), which are coexpressed as complementary partners, and are known biomarkers for hepatic apoptosis and steatohepatitis^18^. Simple epithelia, as found in the liver, pancreas, and intestine, generally express multiple keratins (keratins 7, 8, 18–20), but adult hepatocytes are unique in only expressing K8 and K18^19^. The main function of keratins is protection from cellular stress, with various human diseases, being caused (or predisposed to) by mutations in the intermediate filament proteins^20^,^21^. In cancer, K18 is thought to modulate not only Fas and tumor necrosis factor signaling, but also several intracellular signaling pathways, and operates in conjunction with various related proteins^22^. In addition, phosphorylated K8 has a high reactivity for being crosslinked by TG2, leading to Mallory-Denk body (MDB) formation^8^,^23^.

In this study, we evaluated the enhanced activity of each TG isozyme, and globally identified the Lys-donor substrates for each TG isozyme and the Gln-donor substrates for the entire TG family during the induction of liver fibrosis. This showed that the modification of K18 and K8 via crosslinking by TGs might be involved in liver fibrosis and in the accompanying hepatocytes apoptosis. These results provide a novel insight into the mechanisms of tissue fibrosis, identify a useful target for antifibrotic therapy, and will also be helpful in elucidating the physiological and pathological functions of TGs.

## Results

### Evaluation of fibrotic markers and the expression of TG isozymes during liver fibrosis

**Figure 1:**
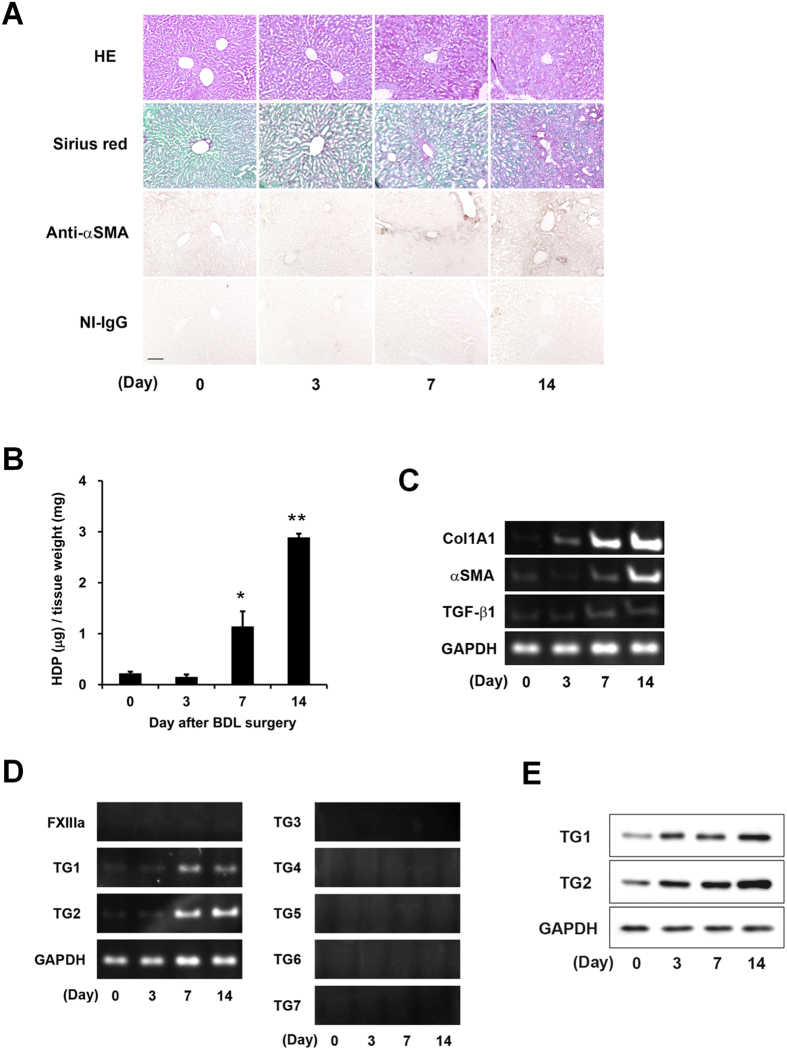
Evaluation of the fibrotic markers and expressions of the transglutaminase (TG) isozymes during liver fibrosis. To induce liver fibrosis, the common bile duct was double-ligated and cut between the ligatures in 8 weeks-old ICR mice. The mice were then sacrificed on days 3, 7, and 14 after surgery (n = 3 mice). (**A**) Liver sections were fixed in 4% paraformaldehyde, and then stained using hematoxylin and eosin (HE), Sirius Red (using the Sirius Red Collagen Detection Kit), and immunohistochemistry (using anti-α smooth muscle actin (αSMA) antibody and rabbit non-immune immunoglobulin G (NI-IgG) as the negative control). The red and green colors indicate the fibrillar collagen (type I to V collagen) and non-collagenous protein, respectively. Bar = 100 μm. (**B**) Hydroxyproline (HDP) contents were evaluated in the liver on each indicated day after bile duct ligation (BDL). (*P < 0.05; **P < 0.01) (**C** and **D**) The mRNA expression levels of the fibrotic markers (Collagen Iα1 (ColA1), αSMA, transforming growth factor (TGF)-β1, GAPDH and the TG family (FXIIIa and TG1–7) were confirmed by RT-PCR. The successful detections for TG3–7 using each primer pair are confirmed in the other tissue extracts. (**E**) The protein levels in the whole lysate from the liver tissue were analyzed by immunoblotting using each antibody and glyceraldehydes 3-phosphate dehydrogenase (GAPDH) as a loading control for each sample.

Liver fibrosis was induced in mice by bile duct ligation (BDL). To evaluate fibrotic levels, the accumulations of collagen and α smooth muscle actin (αSMA) were measured in the livers at 3, 7, and 14 days after BDL treatment. The Sirius Red staining and immunostaining using anti-αSMA antibody showed a dramatic increase in collagen and αSMA around the periportal area on 7 and 14 days after BDL (Fig. 1A). In addition, hydroxyprolin (HDP) levels increased 5.1-fold at 7 days and 13-fold at 14 days after BDL compared with the control (Day 0; Fig. 1B), and levels of mRNA expression of collagen Iα1, αSMA, and transforming growth factor (TGF)-β1, analyzed by reverse transcription polymerase chain reaction (RT-PCR), were markedly higher in BDL-treated mice (Fig. 1C).

To investigate the expression levels of the TG family members, we confirmed the mRNA values of all TG isozymes (Fig. 1D). The mRNA expressions of TG1 and TG2 were enhanced in the 7 and 14 days after BDL surgery, while the mRNAs of the other TG isozymes were not detectable following a 40-cycle PCR analysis. The enhanced protein levels of TG1 and TG2 were also observed in BDL-treated mice (Fig. 1E).

### Measurement of isozyme-specific TG activities in liver extracts

**Figure 2:**
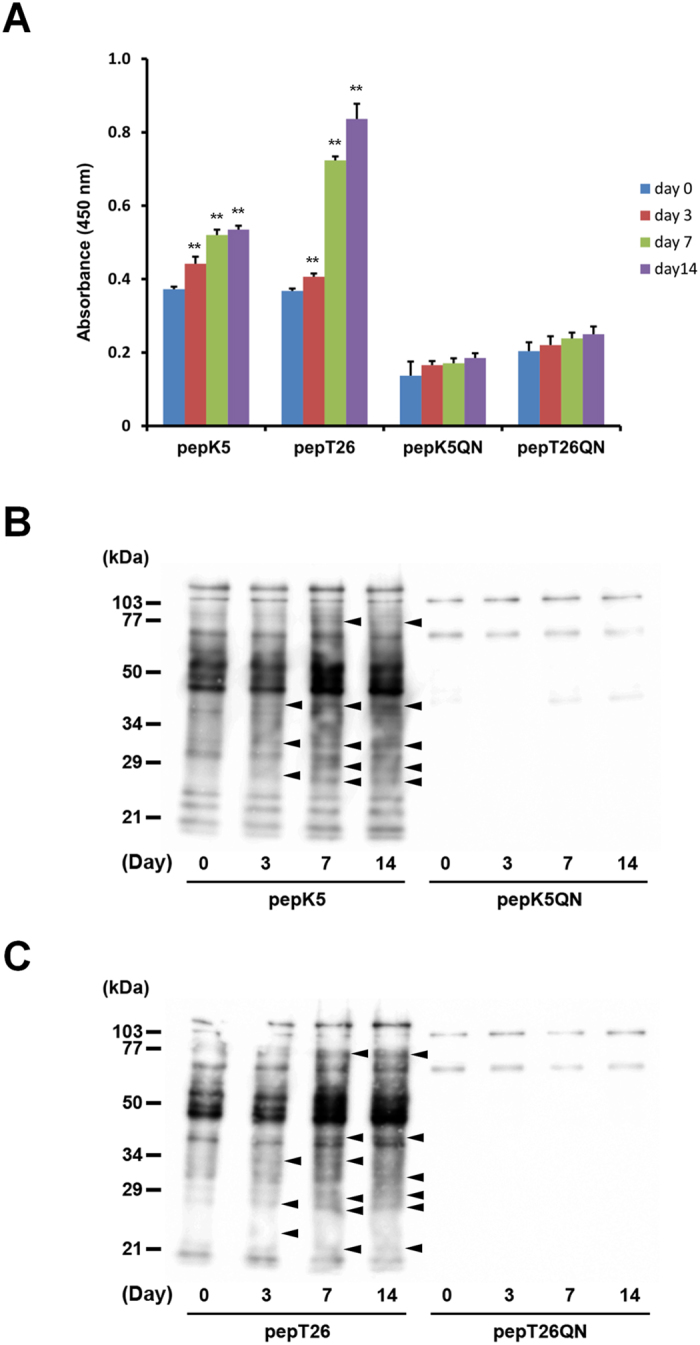
Measurement of isozyme-specific TG activities in liver extracts. Each liver extract was examined for *in vitro* enzymatic activities on the indicated days after BDL surgery (n = 4 mice) using the biotinylated peptides pepK5 (for TG1) and pepT26 (for TG2). Mutant peptides (pepK5QN and pepT26QN) were used as negative controls. (**A**) Each biotinylated peptide was incorporated into β-casein coated on the microtiter wells in the presence of the liver extracts. The amounts of the β-casein that were incorporated with the peptides were measured using peroxidase-conjugated streptavidin. (**P < 0.01) (**B** and **C**) The amount of Lys-donor substrates incorporated with biotinylated peptides on the blotting membrane was detected using peroxidase-conjugated streptavidin. The sizes of the protein mass markers are shown on the left. Arrowheads indicate the bands that increased compared with the control sample (Day 0).

**Figure 3:**
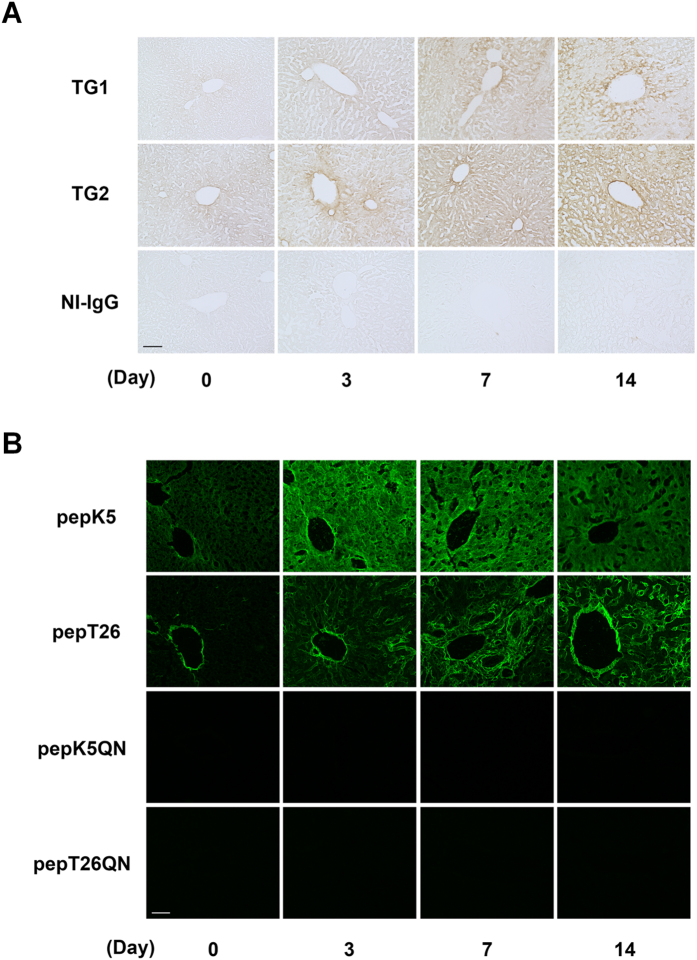
Distributions of the expressions and activities of TG1 and TG2 in fibrotic livers. Each liver section was subjected to the immunohistochemistry and *in situ* TG activity staining on the indicated days after BDL surgery (n = 3 mice). (**A**) Immunostaining was performed using polyclonal anti-mouse TG1 and TG2 antibodies, and rabbit NI-IgG as the negative control. Bar = 100 μm. (**B**) The *in situ* activities of TG1 and TG2 were visualized using FITC-labeled substrate peptides (pepK5 and pepT26, respectively). Mutant peptides (pepK5QN and pepT26QN) were used as the negative control. Bar = 50 μm.

To further investigate TG activities during fibrosis, we analyzed the *in vitro* and *in situ* activities (Figs 2 and 3B). In our system, the isozyme-specific activities of TG1 and TG2 could be measured using the substrate peptides pepK5 and pepT26^14^,^15^. To assess TG activities, we used biotinylated substrate peptides and their mutant forms (pepK5QN and pepT26QN) in which Gln was replaced with an asparagine. These biotinylated peptides were incubated in β-casein coated microtiter wells and the amount of peptide that was incorporated into the β-casein by endogenous TGs in the liver extracts were evaluated using peroxidase-conjugated streptavidin. The results indicated that both TG1 and TG2 activities significantly increased in a time-dependent manner, following BDL treatment (Fig. 2A).

Liver extracts were then incubated with each biotinylated peptide, and resultant proteins that incorporated the peptide were subjected to SDS-PAGE and blotted to a membrane, then detected using peroxidase-conjugated streptavidin (Fig. 2B and C). These yielded results consistent with those shown in Fig. 2A, whereby both TG1 and TG2 activities were marginally enhanced, and the several possible Gln acceptor substrates incorporating each peptide were increased in fibrotic livers as indicated with arrowhead.

### Distribution of TG1 and TG2 expressions and activities in fibrotic liver

To evaluate the distributions of the increased TGs activities, we immunostained liver sections obtained at set days after BDL (Fig. 3A). The signals for both TG1 and TG2 expressions were higher in the periportal area in the early fibrotic stage (Day 3) and across a widespread area in the relatively late fibrotic stages (Days 7 and 14), compared with the control. We then visualized the *in situ* enzymatic activities using fluorescein isothiocyanate (FITC)-labeled substrate peptides in the unfixed liver sections (Fig. 3B). This indicated that TG1 activity was significantly enhanced over a widespread area of the fibrotic liver, while increases in TG2 activity were limited to the periportal area in the early fibrotic stage and to the extracellular space in the late fibrotic stages. It seems that TG1 activity was enhanced faster than TG2 activity. Although fluorescence intensity was measured, no significant difference was observed between the enhanced activity of TG1 and TG2 (Supplementary Fig. S1). The combined pattern of these TG1 and TG2 activities was observed by incorporating BPA (Supplementary Fig. S2).

**Figure 4:**
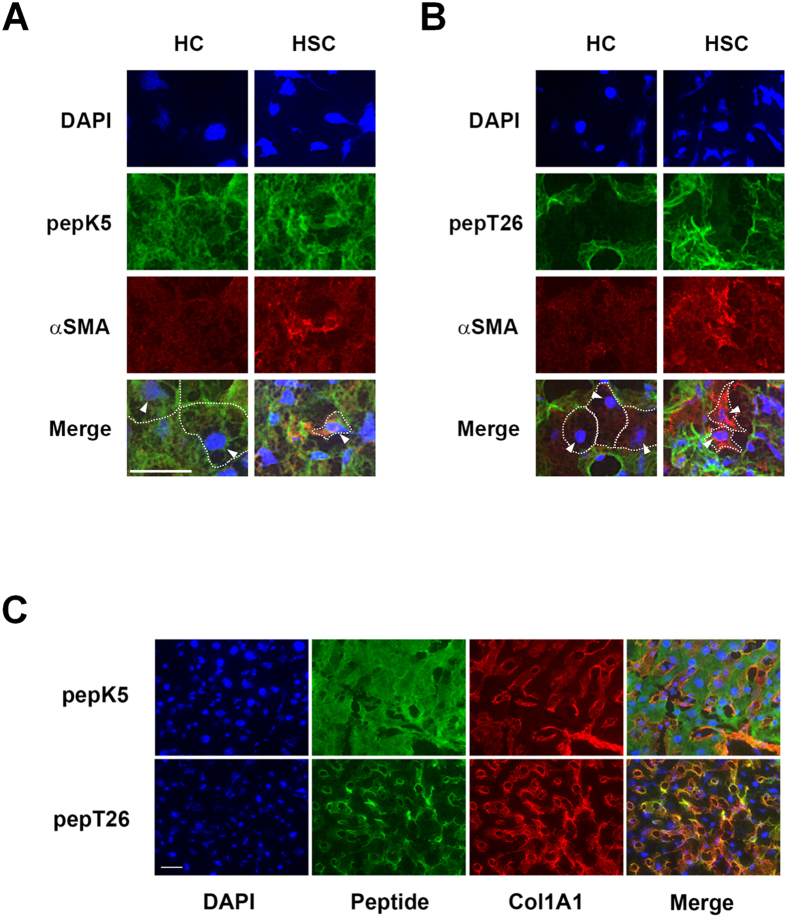
Detailed distribution analysis of the enhanced activity of each TG. The colocalization of the activity of each TG with the αSMA or collagen in the liver sections was analyzed in the control and at 14 days after BDL surgery. (**A** and **B**) The liver sections (n = 3 mice) were incubated with FITC-labeled substrate peptides. Following fixation in 4% paraformaldehyde, the sections were immunostained using anti-αSMA antibody and counterstained using DAPI. Merged staining images are shown in the bottom lane, with arrowhead indicating the nucleus of hepatocyte (HC) and activated hepatic stellate cell (HSC). White dotted lines outline the shape of HC and HSC. Arrowheads indicate the position of the nuclei in these cells. Bar = 50 μm. (**C**) Merged images for the colocalization of the activity of each TG and collagen deposition are shown. Bar = 50 μm.

To further evaluate the distribution of each TG activity in detail, we analyzed their colocalization with αSMA and collagen (Fig. 4). This showed that TG1 activity was enhanced in both hepatocyte cell (HC) and αSMA-positive activated hepatic stellate cell (HSC), whereas the majority of TG2 activity occurred in the extracellular space (Fig. 4A and B). In addition, the expression of collagen was colocalized with TG2 activity but not TG1 activity (Fig. 4C).

### Effect of a TG inhibitor during liver fibrosis

**Figure 5:**
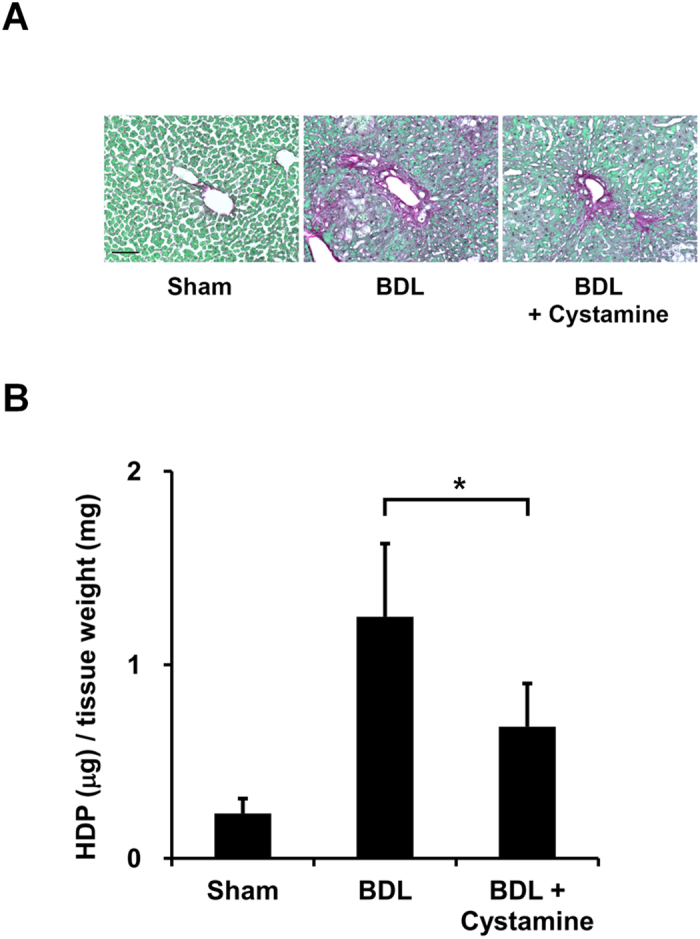
Effect of a TG inhibitor during liver fibrosis. Cystamine, which is a competitive inhibitor for the crosslinking activity of TGs, was orally administrated in drinking water (approximately 8.75 mg/kg/day) from 2 days before the BDL surgery (n = 4 mice). At 14 days after BDL, the fibrotic livers were collected and evaluated for collagen deposition. (**A**) The liver sections were stained using a Sirius Red Collagen Detection Kit. The red and green colors indicate the fibrillar collagen (type I to V collagen) and non-collagenous protein, respectively. Bar = 100 μm. (**B**) The HDP contents were also evaluated in the same liver sections. (*P < 0.05).

To confirm whether the crosslinking activities of TGs induce fibrosis in the liver, cystamine, a competitive inhibitor of TG activity, was orally administered to BDL-treated mice (Fig. 5). As anticipated, cystamine treatment reduced collagen deposition at 14 days after BDL, as indicated by Sirius Red staining (Fig. 5A) and the HDP content (Fig. 5B). Since TG2 knockout mice showed similar levels of liver fibrosis after BDL surgery as wild-type mice (Supplementary Fig. S3), these findings suggest that isozymes other than TG2 play an important role in fibrosis induction.

### Identification of the crosslinked proteins incorporated with each substrate peptide

Each substrate peptide was incorporated into various proteins in the fibrotic livers (Figs 3B and 4). Therefore, we identified the proteins incorporated in these substrate peptides. Each biotinylated substrate peptide was incubated with the liver extracts, and the peptide-incorporated proteins were then purified using monoavidin gel and subjected to trypsin digestion. The fragmented peptides were fractionated by nano-HPLC and identified using MALDI-TOF/TOF mass spectrometer. The resulted in 43 and 42 of the possible substrates for TG1 and TG2, respectively, that contained at least one reactive Lys residue being identified only in the fibrotic liver and not in the untreated control liver (Tables 1 and 2; Supplementary Tables S1 and S2). In addition, a total of 65 unique proteins incorporating BPA that contained at least one Gln residue were also identified as TG substrates (Supplementary Tables S3 and S4). Interestingly, the fibrotic marker fibronectin were included among the possible substrates identified for TG2 but not for TG1.

To examine the time dependency of the reaction, four proteins were identified as possible TG1 substrates (keratin 18, catalase, histone H4 and tubulin α-1C; Table 1) and TG2 substrates (keratin 18, keratin 8, dehydrogenase/reductase SDR family member 4, and histone H2B; Table 2). The identified proteins were annotated with Gene Ontology information from the Uniprot database using Gene Ontology Consortium (http://geneontology.org/) and Protein Analysis Through Evolutionary Relationships (PANTHER; http://pantherdb.org/). This resulted in the substrates for TG1 being categorized into six protein classes (tubulin, histone, peroxidase, intermediate filament, structural protein, and ribosomal protein) and those for TG2 being categorized into three protein classes (histone, ribosomal protein, and actin family cytoskeletal protein). Most proteins were linked to molecular functions involved in structural constituent of cytoskeleton and ribosome. However, interestingly, only the TG1 substrates included proteins with peroxidase and oxidoreductase activity. In terms of biological processes, the identified TG1 and TG2 substrates were involved in chromatin organization regulating the cellular activities via various gene expressions and cellular component morphogenesis relating to the generation and organization of cellular structures.

### Detection and analysis of crosslinked substrates involved in the induction of liver fibrosis

**Figure 6:**
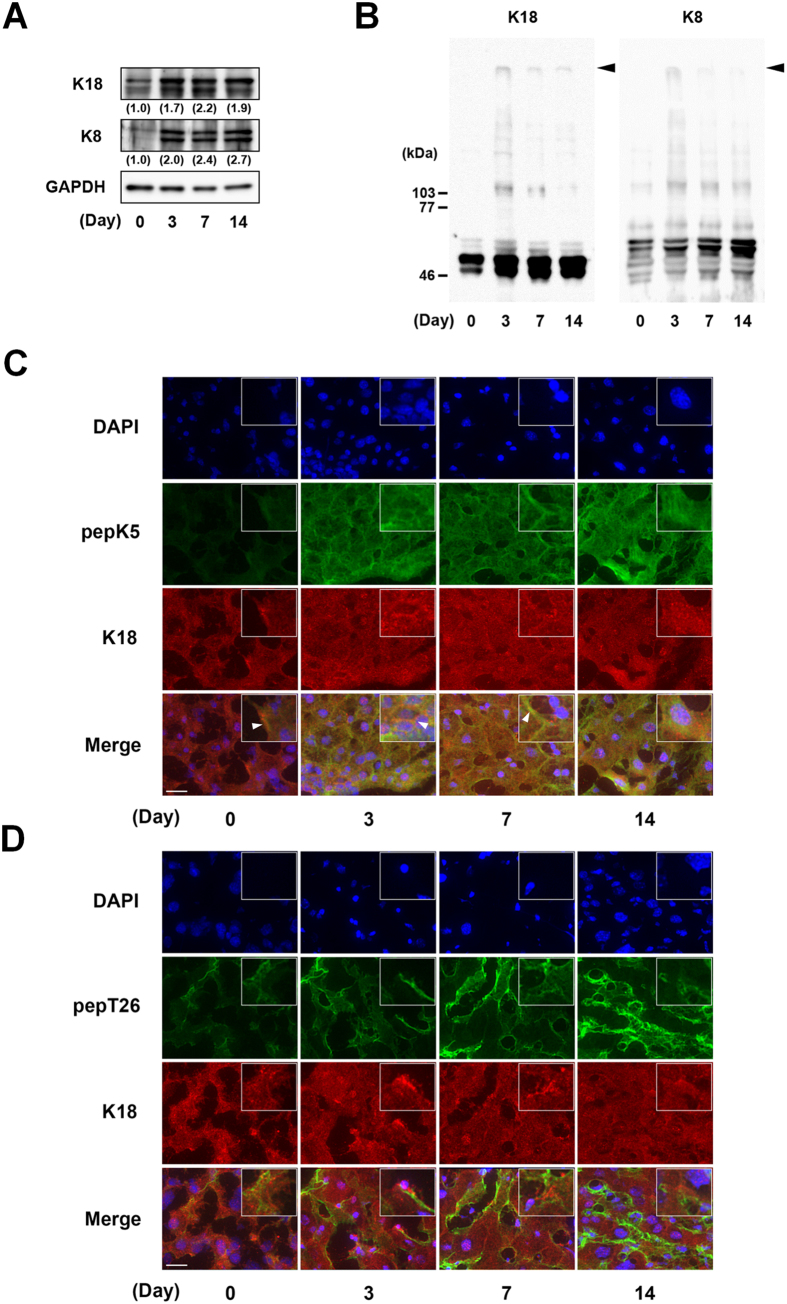
Detection and analysis of crosslinked substrates relating to the induction of liver fibrosis. The expressions of K18 and K8 (48 kDa and 55 kDa as deduced molecular weights, respectively) were evaluated in whole tissue extracts (**A**) and high salt extracts (**B**) by SDS-PAGE and immunoblotting analysis using each polyclonal antibody and GAPDH as a loading control for each sample. Relative changes in the densitometric profiles of K18 and K8 levels are presented under corresponding bands after normalizing to the changes in GAPDH. The arrowheads in B indicate the K18 and K8 proteins of high molecular weight in the gel top, as are found in inclusion bodies. (**C** and **D**) The colocalization of the activity of each TG with K18 in the liver sections was analyzed in the control and at 3, 7, and 14 days after BDL surgery. The liver sections were incubated with FITC-labeled substrate peptides and fixed in 4% paraformaldehyde, following which they were immunostained using anti-K18 antibody plus Alexa 594 anti-rabbit IgG and counterstained using DAPI. Merged staining images are shown in the bottom lanes. The boxed area in inset is 4-fold higher magnification image. The arrow heads indicate the similar distribution of the activity of each TG with K18. Bars = 50 μm.

Liver fibrosis is caused by a dynamic process of ECM, which accompanies morphological changes in several types of cells, including vascular cells, HC, and HSC under the regulation of various gene expressions. Among the identified substrates for TG1 and TG2, both K18 and K8 are known to be involved in liver disease, and are biomarkers for hepatic apoptosis and steatohepatitis^18^. Therefore, we next evaluated the expression and distribution of these proteins in the fibrotic liver (Fig. 6). The expressions of K18 and K8 were enhanced in the fibrotic liver when total cell extracts (Fig. 6A) and high salt extracts (Fig. 6B) were used to detect these high molecular weight proteins as shown in the inclusion body such as MDB^8^,^23^. The expressions of K18 and K8 were distributed across a widespread area of the liver although the relative strong signals of K18 and K8 appeared to be partly localized along a plasma membrane especially in fibrotic liver. Interestingly, these distributions were partly similar with the enhanced activity of TG1 but not of TG2 as indicated with arrowhead in the boxed higher magnification images in inset (Fig. 6C and D; Supplementary Figs S4 and S5).

## Discussion

Posttranslational modifications by TGs, such as the formation of crosslinks between proteins, deamidation, and the attachment of primary amines, are important for various biological processes. The eight isozymes that constitute the TG family are widely distributed across specific tissue types and cells, where they are involved in multiple biological processes. TG1, TG2, and FXIII have been identified as the major isozymes, and thus, their structures, expression patterns, substrates, activities, and relationships with diseases have been investigated.

Most previous studies on the liver have limited their attention to analyzing TG2^5^,^6^,^7^,^8^,^9^,^10^,^11^,^12^,^13^. However, in this study, the mRNA and protein expressions of TG1 also markedly increased during liver fibrosis (Fig. 1D and E). Because only a low level of TG1 expression was observed when a lower concentration of detergent was used (data not shown), it appears that TG1 is anchored to the inner surface of the plasma membrane, as seen in keratinocyte^24^, and may exert its crosslinking activity over a broad area via activation by limited digestion during the induction of liver fibrosis.

Liver fibrosis was induced by BDL, which is commonly used as a model of experimental hepatic fibrosis. Collagen Iα1 and αSMA expressions were markedly higher 7 and 14 days after BDL surgery (Fig. 1C). Our results showed that the expression of TGF-β1 was moderate compared with that of other fibrotic markers. Because TGF-β1 is mostly produced in a latent form, it might be appropriate to measure the levels of active TGF-β1 rather than the transcript. Among all the TG isozymes, mRNA expression of TG1 and TG2 specifically increased in fibrotic liver (Fig. 1D). To exhaustively assess the role of the TG family members, we evaluated the distributions of their expression and enzymatic activities. Previously, labeled primary amines, such as BPA have been used to measure TG activities by evaluating the amount incorporated into Gln-donor substrates. However, this does not allow the TG activity of specific isozymes to be identified. Therefore, here, we used the specific substrate peptide for TG1 and TG2 to evaluate the variation in isozyme-specific TG activities during the induction of liver fibrosis. This showed that the activities of TG1 and TG2 significantly increased in a time-dependent manner following BDL surgery (Fig. 2). Furthermore, the use of fluorescence-labeled substrate peptides showed that, in the early stage of liver fibrosis (Day 3), *in situ* TG1 activity was markedly enhanced across a widespread area of the liver, whereas TG2 activity increased only in the periportal area (Fig. 3B); in the late stage of liver fibrosis (Day 7 and 14), TG1 activity was distributed throughout the cells, including HC and HSC, whereas TG2 activity was localized in the extracellular space where it was colocalized with collagen (Figs 3B and 4). These findings suggest that TG1 plays a key role in the modification of intracellular substrates via crosslinking, whereas extracellular TG2 contributes to the stabilization and maturation of fibrotic proteins, such as collagen and fibronectin. Immunostaining analysis showed that TG1 expressions partly correlate with these activity profiling (Fig. 3A and B). In these cases, there may have been some activation mechanisms involved, such as binding of activator and limited proteolysis^25^,^26^.

The identification of possible TG1/TG2 substrates and the observation of colocalization with the newly identified substrates K18 and K8 supported our hypotheses. The extracellular fibrotic marker fibronectin was among the possible substrates identified for TG2 but not TG1 (Tables 1 and 2). In addition, the intracellular anti-apoptotic protein K18 revealed partly similar distribution with the enhanced activity of TG1 compared with that of TG2 (Fig. 6C and D). These findings suggest that intracellular TG1 and extracellular TG2 have a potential role in crosslinking substrates in the fibrotic liver, and that the modification of K18 through crosslinking by TG1 may influence its anti-apoptotic role against hepatocytes in BDL-treated mice. Furthermore, as expected, TG1 and TG2 knockdown marginally reduced the apoptotic levels in hepatocytes treated with glycochenodeoxycholic acid (GCDCA) (unpublished data), which is a bile salt formed in the liver that has a relatively high toxicity and concentration in the bile and serum following cholestasis, extending its use in cellular models of the disease^27^. Further investigations into the detailed mechanisms involved and biochemical analyses of crosslinked K18 and K8 are ongoing.

The TGs are generally thought to exhibit high substrate specificity. Although the same protein substrate is often recognized by different TG isozymes, the reactivity and specificity differ between them^28^. We and previous report mentioned that TG2 knockout mice showed similar levels of liver fibrosis after BDL surgery as wild-type mice (Supplementary Fig. S3)^13^. This is thought to be causally related to the compensation by the other isozymes. TG1 expression was significantly enhanced in the liver from TG2 knockout mice^29^, indicating that TG1 could be functioning to compensate for the decreased transamidating activity in TG2 knockout mice. As anticipated, the TGs inhibitor cystamine reduced liver fibrosis accompanied by decreased collagen deposition (Fig. 5). However, cystamine inhibits TG but not specifically due to off-target effects, and in addition to TG, it also inhibits caspase 3 activity and increases intracellular glutathione levels^30^. This may not only be due to the contribution of other TG isozymes such as TG1 but also because of other effects of cystamine.

TG2 has been shown to participate in both kidney and pulmonary fibrotic processes. TG2 knockout and inhibition of TG2 reduces tubulointerstitial fibrosis, preserves function in experimental chronic kidney diseases^31^,^32^,^33^, and decreases pulmonary fibrosis^34^.

In this study, we globally identified the various possible substrates incorporated with peptides using biotinylated substrate peptides for TG1 and TG2 (Tables 1 and 2). In many articles in research on the enzymes, lower molecular weight substrate molecules such as labeled primary amine (biotinylated pentylamine as glutamine-acceptor substrate) and Gln-containing peptide (as lysine-acceptor substrate) have been commonly used for the identification of possible protein substrates as well as *in vitro* detection of enzymatic activity^15^,^16^,^35^,^36^. Because the identified substrate candidates by this procedure appeared mostly crosslinked components *in vivo*, we believe the identified molecules in this study are highly possible as substrates by our identified substrate peptide. More recently, TG1-specific keratinocyte substrates by the similar method could be successfully identified^37^.

The findings suggested that several possible substrates isozyme-specifically reacted with each peptide in extracts from fibrotic livers, although there was some overlap in the proteins identified as possible substrates for each TG. However, we were unable to identify fibrotic marker proteins other than fibronectin. This is possibly due to these proteins having a tendency to be insoluble as a result of excessive crosslinking, as only soluble proteins can be reacted with a peptide, purified by an affinity resin, and identified using mass spectrometry. In addition, the possible substrate proteins identified were limited to Gln-acceptor substrates because the Lys-donor peptide or substrate has a lower tendency to be specific to each isozyme in the crosslinking reaction. Therefore, the identification of the counterpart is an essential next step using Lys-donor substrates. To test these hypotheses, extracts containing a high concentration of detergent (1% TritonX-100) were reacted with BPA, which, as expected, resulted in collagen being included among the BPA-incorporated proteins in the fibrotic liver (data not shown). These substrate identifications using biotinylated peptide for TG1 and TG2 were performed as initial trial and these results displayed the several overlapping and abundant proteins. To develop a better understanding about the unique substrate for TG1 and TG2, further optimization about the purification and fractionation step of cellular components such as nucleus, membrane, and extracellular proteins are underway in our laboratory.

In conclusion, we determined the enhancement and distribution of the activity of each TG using isozyme-specific substrate peptides in fibrotic livers from BDL-treated mice. In addition, we globally identified the possible proteins that were incorporated with biotinylated substrate peptides for each TG isozyme in fibrotic livers. Among the unique substrates that were identified, we analyzed the expression levels and distributions of K18 and K8, which are related to the induction of hepatic apoptosis. Our findings suggest that the activity of each TG was independently activated in a different area of the liver tissue during fibrotic induction, and played a potential role in the functional modification of substrates such as K18 and K8, which are relevant to liver fibrosis progression.

## Methods

### Materials

Chemical reagents were purchased from WAKO chemicals (Osaka, Japan) and Nacalai Tesk (Osaka, Japan). Rabbit polyclonal anti-α smooth muscle actin (αSMA) and anti-collagen I antibodies were purchased from Abcam (Cambridge, UK), while anti-K18 and anti-K8 antibodies were purchased from Santa Cruz Biotechnology Inc. (Santa Cruz, CA) and Novus Biologicals (CO, USA), respectively. Rabbit polyclonal anti-TG1 and -TG2 sera were made by Japan Lamb (Hiroshima, Japan) using each of the recombinant TGs that was produced in our laboratory as an antigen^38^. IgG was affinity purified using NHS-activated Sepharose 4 Fast Flow, which was immobilized with recombinant protein (GE Healthcare Ltd., Buckinghamshire, UK). Horseradish peroxidase and Alexa 594-conjugated anti-rabbit IgG were obtained from Jackson ImmunoResearch Laboratories (West Grove, PA, USA) and Invitrogen (Carlsbad, CA, USA), respectively. The 5-(biotinamido) pentylamine (BPA), a biotinylated primary amine substrate for the TGs, was obtained from Pierce (IL, USA), while 4′,6-diamidino-2-phenylindole (DAPI) was obtained from Sigma (MO, USA).

### Ethics statement

Animal care and experiments were conducted according to the Regulations for Animal Experiments in Nagoya University. All surgeries were performed under anesthesia and all efforts were made to minimize suffering. This study was approved by the Animal Care and Use Committee of Nagoya University.

### Animal surgery and experimental protocol

Eight- to ten-weeks-old ICR mice were purchased from Japan SLC Inc (Shizuoka, Japan) and housed in groups of 3-4 mice per cage with food and water available *ad libitum*. The surgical laparotomy and common bile duct ligation (BDL) was performed according to the method described by Arias *et al*.^39^. Briefly, the common bile duct was double ligated and cut between the ligatures. Mice on days 0, 3, 7, and 14 after BDL surgery were perfused with PBS to remove the blood in liver under the anesthesia, and pieces of the livers were either fixed in 4% paraformaldehyde for histological examination or frozen immediately in liquid nitrogen and stored for use in other experiments.

### Immunohistochemical analysis

Cryosections from the liver (10 μm) were fixed, treated with 3% H_2_O_2_, incubated with blocking solution (diluted goat serum), and stained with anti-αSMA, TG1, and TG2 antibodies. Staining signals were enhanced using a VECTASTAIN ABC kit and developed with ImmPACT DAB (Vector Laboratories, Burlingame, CA, USA). As a negative control, the primary antibody was replaced with the same amount of rabbit non-immune IgG (NI-IgG) (Sigma; St. Louis, MO, USA). Sections were also stained with hematoxylin and eosin (Leica Microsystems). Collagen was stained using a Sirius Red Collagen Detection Kit (Chondrex, Redmond, USA). Each experiment was conducted in triplicate.

### Measurement of hydroxyproline contents

The hydroxyproline (HDP) contents were measured as described by Reddy *et al*.^40^. Briefly, approximately 30 mg of frozen liver tissue was hydrolyzed in 2 N NaOH at 65 °C for 10 min, and then incubated at 120 °C for 20 min. Following this, the same amount of 6 N HCl was added and the mixture was then incubated at 120 °C for 20 min. This was then mixed with activated charcoal solution (10 mg/ml in 4 N KOH) and a four-fold concentration of acetate-citrate buffer (pH 6.5) containing 1.8 M sodium acetate, 0.5 M citric acid, 0.4 M acetic acid, and 1.7 M sodium hydroxide. Following centrifugation, the supernatant was incubated with 100 mM chloramine T solution at room temperature for 25 min, after which 1 M Ehrlich’s solution was added and the samples incubated at 65 °C for 20 min. The absorbance of the samples was then measured at 560 nm. Data are provided as the mean ± SEM for triplicate measurements from each sample.

### RT- PCR for fibrotic markers and the TG family

Total RNA was prepared from the frozen livers and used for transcription by reverse-transcriptase (TAKARA Bio, Kyoto, Japan). The cDNAs were then mixed with specific primer pairs (summarized in Table 3), following which PCR was performed. The amplified products were analyzed by 2.5% agarose or 5% polyacrylamide gel electrophoresis.

### Western blotting

The mouse livers were homogenized in lysis buffer containing 10 mM Tris-HCl (pH 8.0), 150 mM NaCl, 0.1 mM EDTA, 1% Triton X-100, and protease inhibitor cocktail (Merck Millipore, Darmstadt, Germany). The soluble fraction was then treated with SDS buffer, subjected to SDS-PAGE, and transferred to a polyvinylidene difluoride (PVDF) membrane (Merck Millipore). The membrane was reacted with primary antibody, and the specific signal was detected by the secondary antibody conjugated with peroxidase and chemiluminesence reagent (Thermo Scientific, IL, USA). Each experiment was conducted in triplicate.

### Measurement of isozyme-specific TG activities in microtiter wells

A microtiter plate assay was performed as described previously^14^. Briefly, β-casein (which was used as the Lys donor substrate) was immobilized in 96-well microplates. The enzyme reaction mixture contained each biotinylated peptide (pepK5 and pepT26 for TG1 and TG2, respectively) in a reaction buffer (final concentration: 20 mM Tris-HCl (pH 8.0), 140 mM NaCl, 2.5 mM dithiothreitol, 15 mM CaCl_2_) and was incubated with 10–20 μg of each liver extract at 37 °C for 20 min. The wells were then washed and the amounts of protein incorporated with biotinylated peptides were measured using peroxidase-conjugated streptavidin (Rockland Immunochemicals, PA, USA) and 3,3′,5,5′-tetramethylbenzidine (Sigma). Data are provided as mean ± SEM for triplicate measurements from each sample.

### *In vitro* and *in situ* detection of TG activities

Liver soluble extract (50 μg) was incubated with 100 μM of each biotinylated peptide (pepK5 and pepT26) in the presence of 5 mM CaCl_2_. The reaction products were then subjected to SDS-PAGE and blotted on a PVDF membrane. The biotinylated peptide-incorporated proteins were detected using peroxidase-conjugated streptavidin and a chemiluminescence reagent. Mutant peptides in which Gln residues were exchanged with asparagine residues (pepK5QN and pepT26QN) were used as negative controls.

The *in situ* TG activity was visualized using FITC-labeled peptides from the unfixed liver sections, as reported previously^16^. The frozen specimen block was cut into 10-μm-thick sections using cryomicrotome (Leica Microsystems, Wetzlar, Germany). These sections were collected using cryofilm, and then incubated in a reaction mixture containing 100 mM Tris-HCl (pH 8.0), 1 mM dithiothreitol (DTT), and 5 mM CaCl_2_ in the presence of 5 μM FITC-labeled peptide at 37 °C for 1 h. Following this, the sections were washed and observed under a fluorescence microscope (BZ-9000; Keyence, Osaka, Japan). The signal intensities in the images were adjusted to maintain linearity using imaging software (Adobe Photoshop CS). Each experiment was conducted in triplicate.

### Identification of candidate substrates

Liver extracts were incubated with each biotinylated substrate peptide (pepK5 and pepT26) and pentylamine, following the same procedure as outlined above for the *in vitro* detection of activity. Following the crosslinking reaction by the endogenous enzymes in the extracts, these samples were applied to SoftLink™ Soft Release Avidin Resin (Promega, WI, USA). The biotinylated proteins were then eluted with 5 mM biotin and precipitated with 10% trichloroacetic acid (TCA)/acetone, following which the samples were dissolved in 8 M urea and mixed with 5 μl of 0.2% Max surfactant (Promega)/50 mM NH_4_HCO_3_ in a vortex mixer. Then, 14.6 μl of 50 mM NH_4_HCO_3_ and 1.25 μl of 0.1 M DTT were added, and the mixture was incubated at 56 °C for 20 min.

For the alkylation of the samples, 1.5 μl of 0.3 M iodoacetamide was added and the samples were trypsinized in the presence of 0.01% Max surfactant. These samples were then fractionated using a reverse-phase Dina Nano-HPLC in a C18 column (KYA Technologies, Tokyo, Japan). Each fraction was mixed with α-cyano-4-hydroxycinnamic acid and spotted on MALDI plate. MALDI-TOF mass spectrometry was then performed using a 5800 Proteomics Analyzer (ABSCIEX, Tokyo, Japan). Mass spectrometry and tandem mass spectrometry (MS/MS) data for each peptide were analyzed using Protein pilot™ software (ABSCIEX).

### High salt extraction for detecting inclusion bodies

High salt extraction was performed according to a previously described protocol^41^. Briefly, liver samples were homogenized in Triton X-100 buffer containing 5 mM EDTA, 1% TritonX-100, and protease inhibitor cocktail (Merck Millipore). The samples were centrifuged and the pellets homogenized in high salt buffer containing 10 mM Tris-HCl (pH 7.5), 140 mM NaCl, 1.5 M KCl, 5 mM EDTA, 0.5% Triton X100, 1 mM phenylmethylsulfonyl fluoride, and protease inhibitor cocktail, mixed at 4 °C for 30 min, and re-pelleted. The pellets were then washed in 5 mM EDTA/PBS buffer, centrifuged to form a pellet, and re-suspended in SDS sample buffer. Samples were separated on a 7.5% SDS-PAGE, following which Western blotting was performed using anti-K18 and anti-K8 antibodies.

## Additional Information

**How to cite this article:** Tatsukawa, H. *et al*. Global identification and analysis of isozyme-specific possible substrates crosslinked by transglutaminases using substrate peptides in mouse liver fibrosis. *Sci. Rep.* **7**, 45049; doi: 10.1038/srep45049 (2017).

**Publisher's note:** Springer Nature remains neutral with regard to jurisdictional claims in published maps and institutional affiliations.

## Supplementary Material

Supplementary Information

## Figures and Tables

**Table 1:** Identified possible substrates for TG1 using pepK5.

Accession number	Name	Days			
		0	3	7	14
P05784	Keratin, type I cytoskeletal 18		+	+	+
P24270	Catalase		+	+	+
P62806	Histone H4		+	+	+
P68373	Tubulin α-1C chain		+	+	+
Q8K0E8	Fibrinogen β chain		+	+	
Q61781	Keratin, type I cytoskeletal 14		+		+
Q8VDD5	Myosin-9			+	+
Q60597	2-oxoglutarate dehydrogenase, mitochondrial		+		
Q9CZX8	40 S ribosomal protein S19		+		
P62849	40 S ribosomal protein S24		+		
P97351	40 S ribosomal protein S3a		+		
P62702	40 S ribosomal protein S4, X isoform		+		
Q6ZWV3	60 S ribosomal protein L10		+		
P47963	60 S ribosomal protein L13		+		
P27659	60 S ribosomal protein L3		+		
P56391	Cytochrome c oxidase subunit 6B1		+		
Q64459	Cytochrome P450 3A11		+		
Q80XN0	D-β-hydroxybutyrate dehydrogenase, mitochondrial		+		
Q9DCW4	Electron transfer flavoprotein subunit β		+		
Q8VCM7	Fibrinogen γ chain		+		
P01942	Hemoglobin subunit α		+		
P43274	Histone H1.4		+		
Q6GSS7	Histone H2A type 2-A		+		
O88844	Isocitrate dehydrogenase [NADP] cytoplasmic		+		
Q9QWL7	Keratin, type I cytoskeletal 17		+		
Q9WVL0	Maleylacetoacetate isomerase		+		
Q91VS7	Microsomal glutathione S-transferase 1		+		
Q5SX40	Myosin-1		+		
P35700	Peroxiredoxin-1		+		
O08709	Peroxiredoxin-6		+		
Q80U40	REVERSED RIMS-binding protein 2		+		
P05367	Serum amyloid A-2 protein		+		
P84104	Splicing factor, arginine/serine-rich 3		+		
Q8BMS1	Trifunctional enzyme subunit α, mitochondrial		+		
Q9CWF2	Tubulin β-2B chain		+		
Q63836	Selenium-binding protein 2		+		
Q9Z1T2	REVERSED Thrombospondin-4			+	
Q9D2U9	Histone H2B type 3-A				+
Q9D646	Keratin, type I cuticular Ha4				+
Q9Z2K1	Keratin, type I cytoskeletal 16				+
Q9Z2T6	Keratin, type II cuticular Hb5				+
Q921I1	Serotransferrin				+
P68372	Tubulin β-2C chain				+

Liver extract on each indicated day after BDL surgery (n = 3 mice) was incubated with biotinylated pepK5. The peptide-incorporated proteins were then purified using monoavidin gel and subjected to trypsin digestion. The fragmented peptides were fractionated by nano-HPLC and identified using MALDI-TOF/TOF mass spectrometer. The newly identified possible substrates in each indicated day were demonstrated as “+ ” compared to control sample (Day 0). The underlined possible substrates indicate overlapped substrates identified as both peptide (pepK5 and pepT26)-incorporated proteins.

**Table 2:** Identified possible substrates for TG2 using pepT26.

Accession number	Name	Days			
		0	3	7	14
P05784	Keratin, type I cytoskeletal 18		+	+	+
P11679	Keratin, type II cytoskeletal 8		+	+	+
Q99LB2	Dehydrogenase/reductase SDR family member 4		+	+	+
Q9D2U9	Histone H2B type 3-A		+	+	+
Q9JJN0	DNA polymerase η		+	+	
Q6ZWV3	60 S ribosomal protein L10		+		+
Q8K0E8	Fibrinogen β chain		+		+
Q8VCM7	Fibrinogen γ chain		+		+
P02089	Hemoglobin subunit β-2		+		+
Q8R0W0	Epiplakin			+	+
P11276	Fibronectin			+	+
O08573	Galectin-9			+	+
Q8VDD5	Myosin-9			+	+
P21981	Protein-glutamine γ-glutamyltransferase 2			+	+
Q921I1	Serotransferrin			+	+
P99024	Tubulin β-5 chain			+	+
P68134	Actin, α skeletal muscle		+		
P26040	Ezrin		+		
Q8R1M2	Histone H2A.J		+		
P17897	Lysozyme C-1		+		
Q5SX40	Myosin-1		+		
Q921X9	Protein disulfide-isomerase A5		+		
P07724	Serum albumin		+		
Q64459	Cytochrome P450 3A11			+	
P68040	Guanine nucleotide-binding protein subunit β-2-like 1			+	
Q8CGP5	Histone H2A type 1-F			+	
P27661	Histone H2A.x			+	
Q8CGP1	Histone H2B type 1-K			+	
Q02257	Junction plakoglobin			+	
Q8VD63	REVERSED Testis-specific Y-encoded-like protein 4			+	
Q9JJZ2	Tubulin α-8 chain			+	
Q921H8	3-ketoacyl-CoA thiolase A, peroxisomal				+
P35979	60 S ribosomal protein L12				+
P47963	60 S ribosomal protein L13				+
Q9CZM2	60 S ribosomal protein L15				+
P35980	60 S ribosomal protein L18				+
P47911	60 S ribosomal protein L6				+
Q64481	Cytochrome P450 3A16				+
P19639	Glutathione S-transferase Mu 3				+
Q3THW5	Histone H2A.V				+
Q60605	Myosin light polypeptide 6				+
P50431	Serine hydroxymethyltransferase, cytosolic				+

Liver extract on each indicated day after BDL surgery (n = 3 mice) was incubated with biotinylated pepT26. The peptide-incorporated proteins were then purified using monoavidin gel and subjected to trypsin digestion. The fragmented peptides were fractionated by nano-HPLC and identified using MALDI-TOF/TOF mass spectrometer. The newly identified possible substrates in each indicated day were demonstrated as “+ ” compared to control sample (Day 0). The underlined possible substrates indicate overlapped substrates identified as both peptide (pepK5 and pepT26)-incorporated proteins.

**Table 3:** Primer pairs for RT-PCR experiments.

Gene	Forward	Reverse	Product size
***Col1A1***	GAGCGGAGAGTACTGGATCG	TACTCGAACGGGAATCCATC	204
***TGFβ1***	ATACGCCTGAGTGGCTGTCT	GGTTCATGTCATGGATGGTG	192
***αSMA***	ACTGGGACGACATGGAAAAG	AGAGGCATAGAGGGACAGCA	203
***FXIIIa***	TGATTGTCCGCAGAGGGCAG	GGGTAGCGACCAATGAC	105
***TG1***	ATCGTGGTAGTAGCCGACGC	ATGGTCACAGAGTCCGAGGC	138
***TG2***	AGCCGATGATGTGTACCTAG	AGGATTCCATCCTCGAACTG	137
***TG3***	TGGAGAAAGGCAGTGATAG	ACTGGAACCTTCTGGATAC	382
***TG4***	AGTCTGGCGTAGAGGTTATTC	CCTGAGCACCACGGATTG	125
***TG5***	GTTCCATTCTGGCAGGACAC	CCCAGGGCACTGATGCGGAT	133
***TG6***	GCTCTGTGCTTGACCAACCT	TGGGATTCACGCAGGATCTC	112
***TG7***	ACAGGGCAGTTCATTCTGGT	TGGTGAGGGTGATGTGGATA	117
***GAPDH***	ACTGGCATGGCCTTCCGTGT	CCTGCTTCACCACCTTCTTG	109
