# Supplementary material for: Global identification and analysis of isozyme-specific possible substrates crosslinked by transglutaminases using substrate peptides in mouse liver fibrosis
Source: Sci Rep. 2017 Mar 22;7:45049. doi: 10.1038/srep45049 (PMC5361200; doi:10.1038/srep45049)
Supplement: Supplementary Information [file srep45049-s1.pdf]

Supplementary Information

Global identification and analysis of isozyme-specific possible substrates crosslinked by transglutaminases using substrate peptides in mouse liver fibrosis

Hideki Tatsukawa*, Yuji Tani, Risa Otsu, Haruka Nakagawa, and Kiyotaka Hitomi

Supplementary Fig. S1

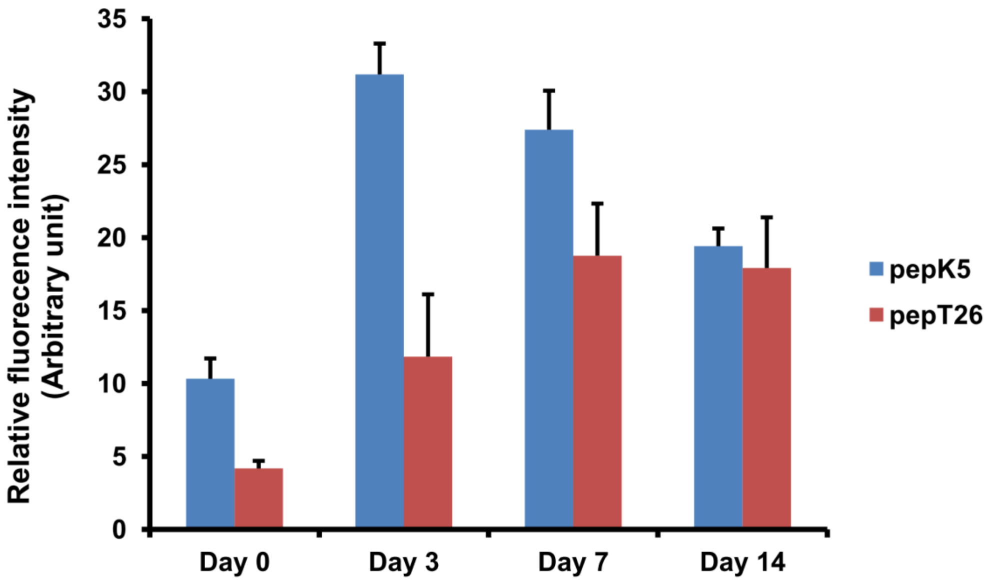

Supplementary Fig. S2

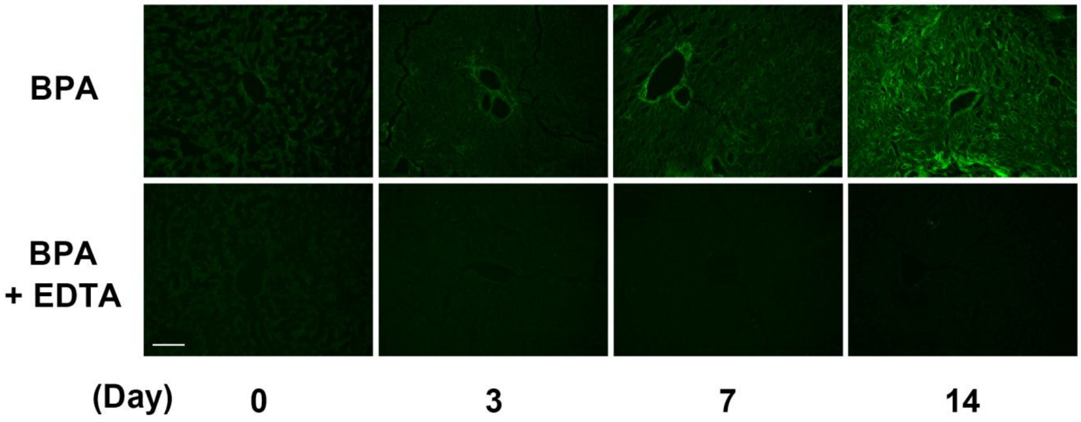

Supplementary Fig. S3

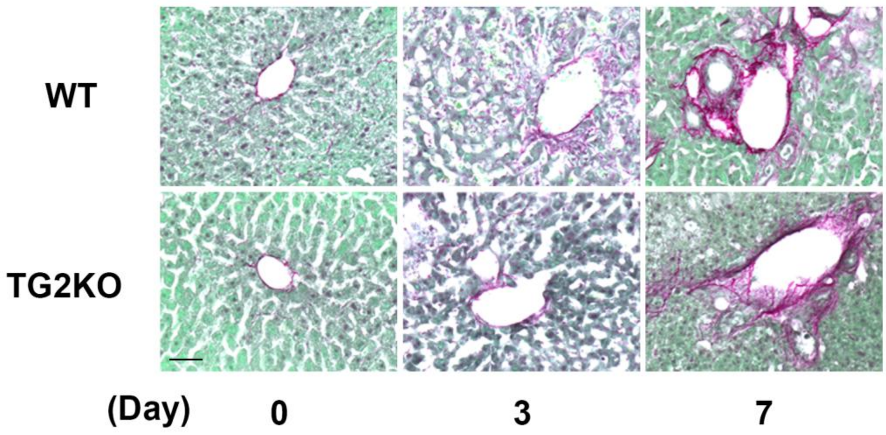

Supplementary Fig. S4

A

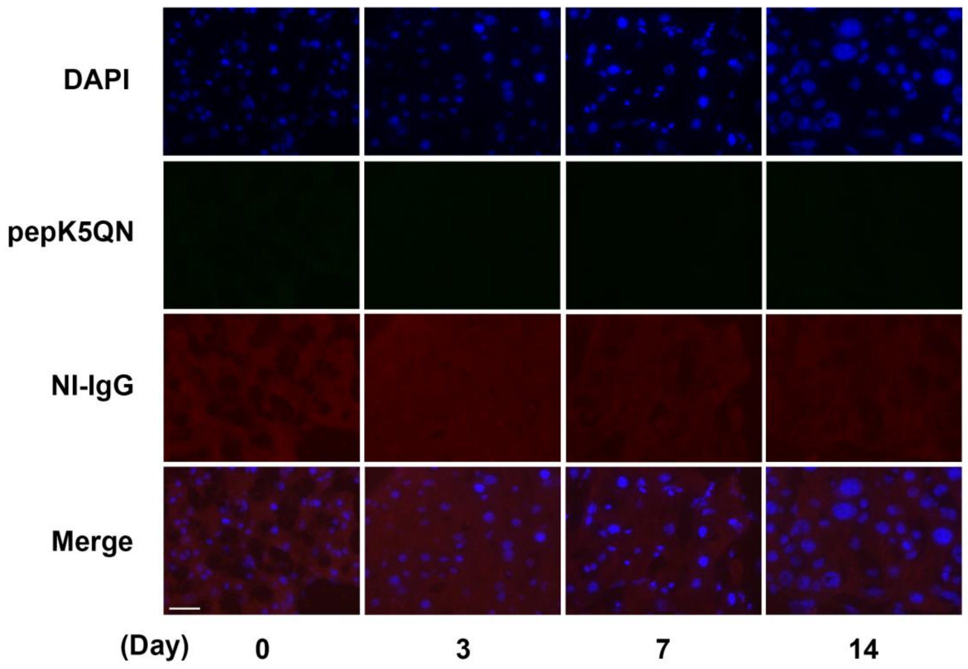

B

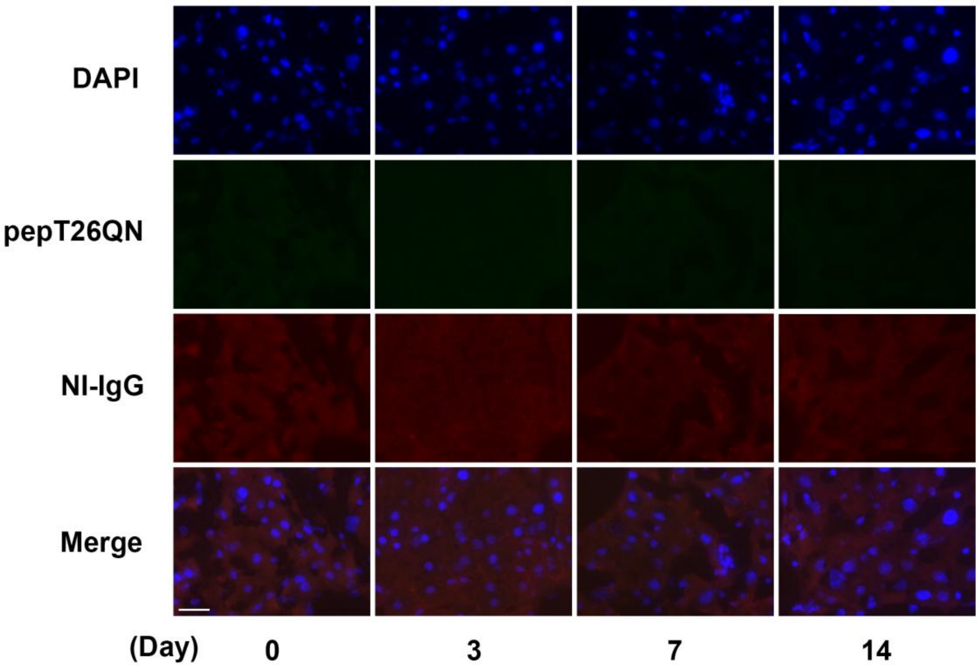

Supplementary Fig. S5

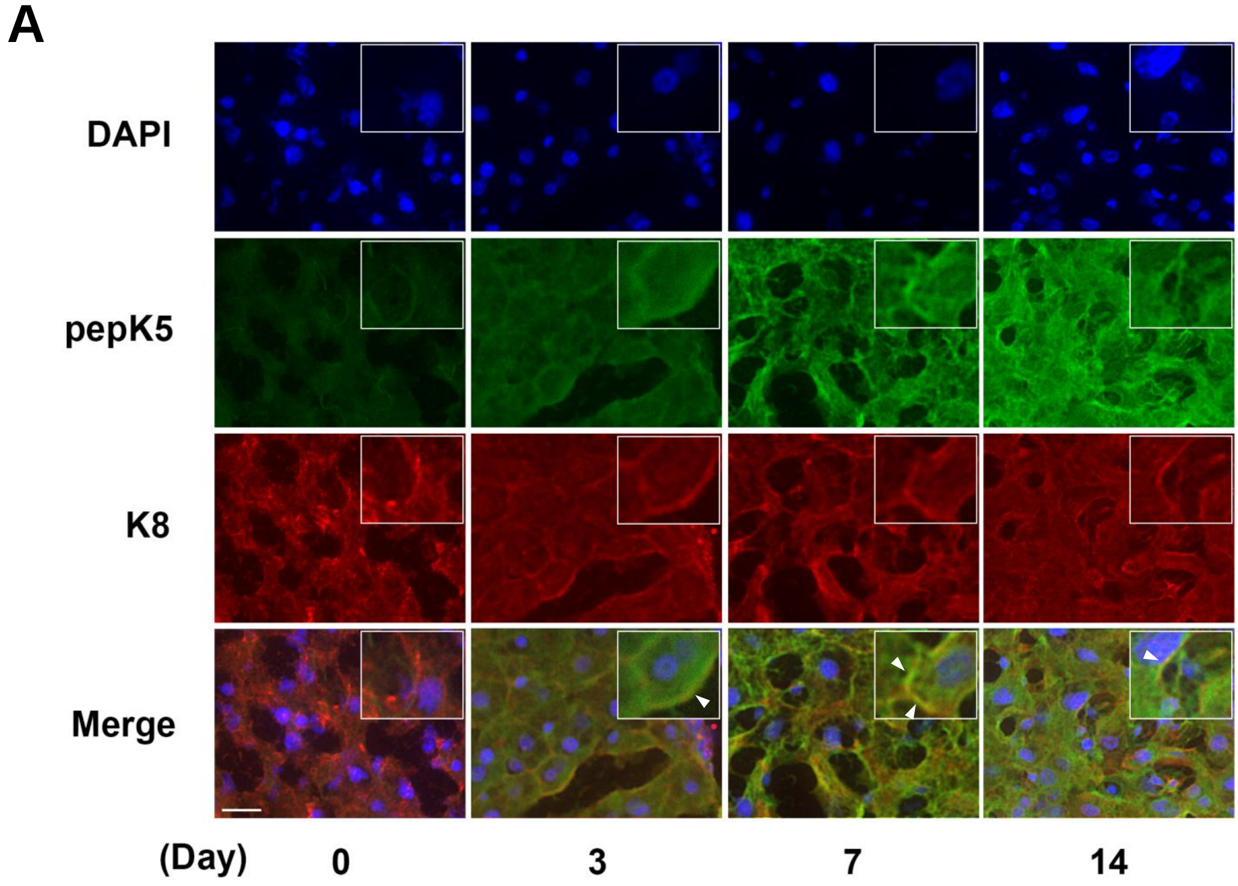

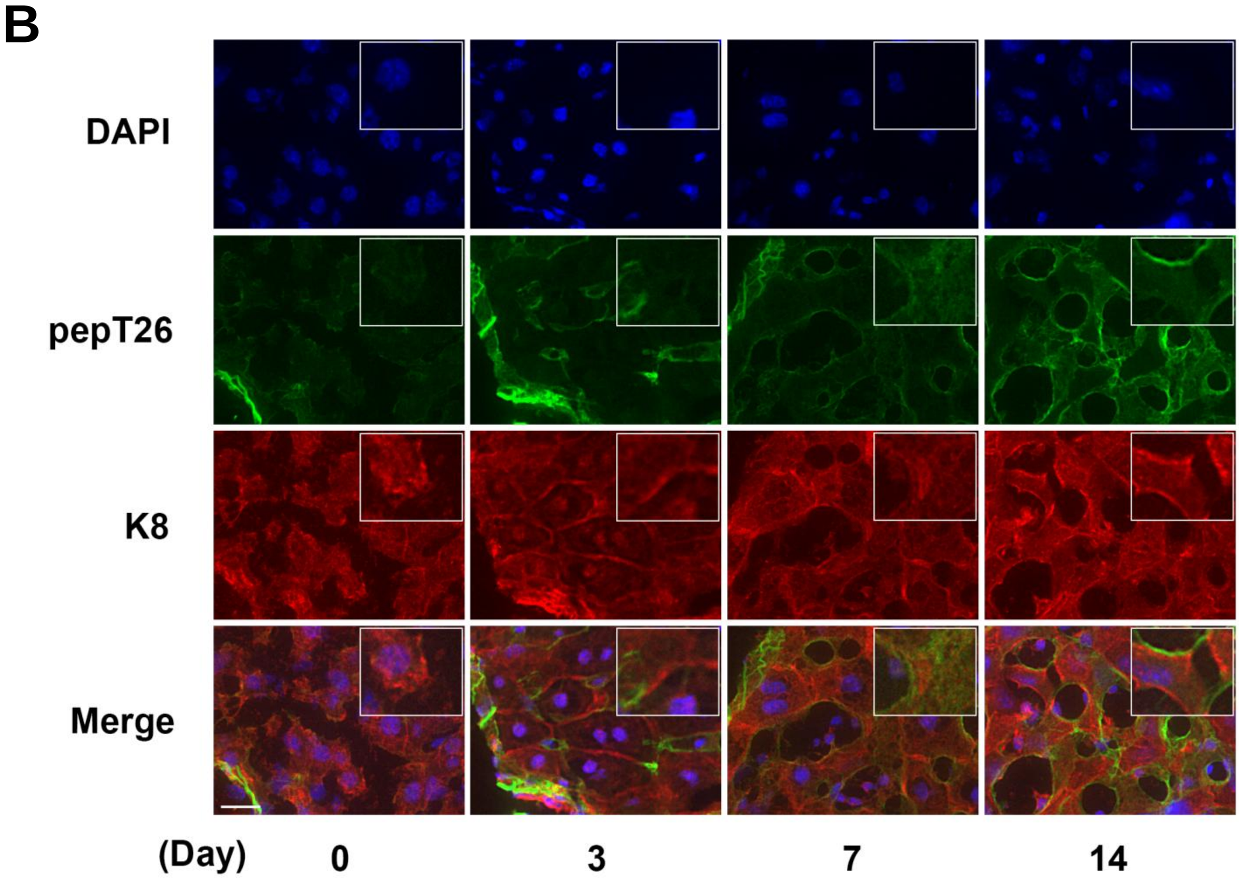

Supplementary Figure Legends

Supplementary Fig. S1 *In situ* measurement of fluorescence intensity in TG1 and TG2 activities in fibrotic liver

Each liver section was subjected to *in situ* TG1 (pepK5) and TG2 (pepT26) activities staining on the indicated days after BDL surgery (Fig. 3B). Fluorescence intensities on at least four different images were measured using the freeware Image J.

Supplementary Fig. S2 Distributions of total TGs activities in the fibrotic liver

Each liver section was subjected to *in situ* TG activity staining on the indicated days after BDL surgery. The total *in situ* TG activities were then visualized using BPA and FITC-labeled avidin. BPA was incubated in the presence of 25 mM EDTA for the negative control. Bar = 100 μm.

Supplementary Fig. S3 Evaluation of collagen deposition in BDL-treated TG2KO mice

At 14 days after BDL, the livers from wild-type and TG2KO mice were collected and evaluated for collagen deposition. The liver sections were stained using a Sirius Red Collagen Detection Kit. The red and green colors indicate the fibrillar collagen (type I to V collagen) and non-collagenous protein, respectively. Bar = 50 μm.

Supplementary Fig. S4 Negative control for the immunostaining in the colocalizaton of K18 with the activity of each TG in fibrotic livers

(A and B) Liver sections were analyzed in the control and at 3, 7, and 14 days after BDL surgery. These were incubated with FITC-labeled QN substrate peptides (pepK5QN and pepT26QN) and then fixed in 4% paraformaldehyde, following which the sections were immunostained using rabbit non-immune IgG plus Alexa 594 anti-rabbit IgG and counterstained using DAPI. Merged staining images are shown in the bottom lanes. Bars = 50 μm.

Supplementary Fig. S5 Colocalizaton of K8 with the activity of each TG in fibrotic livers

(A and B) The colocalization of the activity of each TG with the K8 in the liver sections was analyzed in the control and at 14 days after BDL surgery. The liver sections were incubated with FITC-labeled substrate peptides and then fixed in 4% paraformaldehyde, following which the sections were immunostained using anti-K8 antibody and DAPI. Merged staining images are shown in the bottom lanes. The boxed area in inset is 4-fold higher magnification image. The arrow heads indicate the similar distribution of the activity of each TG with K8. Bars = 50 μm.

Supplementary Table

Supplementary Table S1.

Accession number	Name
Q64433	10 kDa heat shock protein, mitochondrial
Q9CQ62	2,4-dienoyl-CoA reductase, mitochondrial
Q8BWT1	3-ketoacyl-CoA thiolase, mitochondrial
P63038	60 kDa heat shock protein, mitochondrial
P47962	60S ribosomal protein L5
P20029	78 kDa glucose-regulated protein
Q8QZT1	Acetyl-CoA acetyltransferase, mitochondrial
P00329	Alcohol dehydrogenase 1
P47738	Aldehyde dehydrogenase, mitochondrial
Q61176	Arginase-1
P16460	Argininosuccinate synthase
Q03265	ATP synthase subunit α, mitochondrial
P56480	ATP synthase subunit β, mitochondrial
O35490	Betaine--homocysteine S-methyltransferase 1
Q9QZQ8	Core histone macro-H2A.1
Q99LB2	Dehydrogenase/reductase SDR family member 4
P10126	Elongation factor 1-α 1
Q91Y97	Fructose-bisphosphate aldolase B
Q60759	Glutaryl-CoA dehydrogenase, mitochondrial
P16858	Glyceraldehyde-3-phosphate dehydrogenase
O88569	Heterogeneous nuclear ribonucleoproteins A2/B1
P63158	High mobility group protein B1
P10922	Histone H1.0
Q61425	Hydroxyacyl-coenzyme A dehydrogenase, mitochondrial
P11679	Keratin, type II cytoskeletal 8
Q9R0H0	Peroxisomal acyl-coenzyme A oxidase 1
Q9DBM2	Peroxisomal bifunctional enzyme
P51660	Peroxisomal multifunctional enzyme type 2
Q9CY58	Plasminogen activator inhibitor 1 RNA-binding protein
Q921X9	Protein disulfide-isomerase A5

**Q05920**	**Pyruvate carboxylase, mitochondrial**
**Q99PL5**	**Ribosome-binding protein 1**
**P38647**	**Stress-70 protein, mitochondrial**
**Q9D0R2**	**Threonyl-tRNA synthetase, cytoplasmic**
**Q62452**	**UDP-glucuronosyltransferase 1-9**
**P50544**	**Very long-chain specific acyl-CoA dehydrogenase, mitochondrial**

Supplementary Table S1. Identified possible substrates for TG1 in control sample

Liver extract on control sample (Day 0) was incubated with pepK5. The peptide-incorporated proteins were then purified using monoavidin gel and subjected to trypsin digestion. The fragmented peptides were fractionated by nano-HPLC and identified using MALDI-TOF/TOF mass spectrometer.

Supplementary Table S2.

Accession number	Name
Q8R0Y6	10-formyltetrahydrofolate dehydrogenase
Q9CQ62	2,4-dienoyl-CoA reductase, mitochondrial
O08756	3-hydroxyacyl-CoA dehydrogenase type-2
Q8BWT1	3-ketoacyl-CoA thiolase, mitochondrial
P62270	40S ribosomal protein S18
P63038	60 kDa heat shock protein, mitochondrial
P20029	78 kDa glucose-regulated protein
Q8QZT1	Acetyl-CoA acetyltransferase, mitochondrial
P63260	Actin, cytoplasmic 2
Q8VCW8	Acyl-CoA synthetase family member 2, mitochondrial
P50247	Adenosylhomocysteinase
P51881	ADP/ATP translocase 2
P00329	Alcohol dehydrogenase 1
P47738	Aldehyde dehydrogenase, mitochondrial
Q61176	Arginase-1
P16460	Argininosuccinate synthase
Q03265	ATP synthase subunit α, mitochondrial
P56480	ATP synthase subunit β, mitochondrial
O35490	Betaine--homocysteine S-methyltransferase 1
Q8C196	Carbamoyl-phosphate synthase [ammonia], mitochondrial
P16015	Carbonic anhydrase 3
P24270	Catalase
Q9QZQ8	Core histone macro-H2A.1
P56395	Cytochrome b5
P24456	Cytochrome P450 2D10
Q80XN0	D-β-hydroxybutyrate dehydrogenase, mitochondrial
Q99LC5	Electron transfer flavoprotein subunit α, mitochondrial
Q9DCW4	Electron transfer flavoprotein subunit β
P10126	Elongation factor 1-α 1
Q91XD4	Formimidoyltransferase-cyclodeaminase
Q91Y97	Fructose-bisphosphate aldolase B
P26443	Glutamate dehydrogenase 1, mitochondrial
P30115	Glutathione S-transferase A3
P10649	Glutathione S-transferase Mu 1

**P19157**	**Glutathione S-transferase P 1**
**P16858**	**Glyceraldehyde-3-phosphate dehydrogenase**
**Q9QXF8**	**Glycine N-methyltransferase**
**P63017**	**Heat shock cognate 71 kDa protein**
**P11499**	**Heat shock protein HSP 90-β**
**O88569**	**Heterogeneous nuclear ribonucleoproteins A2/B1**
**P62806**	**Histone H4**
**P54869**	**Hydroxymethylglutaryl-CoA synthase, mitochondrial**
**Q922U2**	**Keratin, type II cytoskeletal 5**
**Q63880**	**Liver carboxylesterase 31**
**P41216**	**Long-chain-fatty-acid--CoA ligase 1**
**Q9EQ20**	**Methylmalonate-semialdehyde dehydrogenase [acylating], mitochondrial**
**P32020**	**Non-specific lipid-transfer protein**
**P24369**	**Peptidyl-prolyl cis-trans isomerase B**
**Q9DBM2**	**Peroxisomal bifunctional enzyme**
**P51660**	**Peroxisomal multifunctional enzyme type 2**
**P09103**	**Protein disulfide-isomerase**
**Q05920**	**Pyruvate carboxylase, mitochondrial**
**Q99PL5**	**Ribosome-binding protein 1**
**Q63836**	**Selenium-binding protein 2**
**Q78PY7**	**Staphylococcal nuclease domain-containing protein 1**
**P38647**	**Stress-70 protein, mitochondrial**
**Q9WUM5**	**Succinyl-CoA ligase [GDP-forming] subunit α, mitochondrial**
**Q9D0R2**	**Threonyl-tRNA synthetase, cytoplasmic**
**P50544**	**Very long-chain specific acyl-CoA dehydrogenase, mitochondrial**

Supplementary Table S2. Identified possible substrates for TG2 in control sample

Liver extract on control sample (Day 0) was incubated with pepT26. The peptide-incorporated proteins were then purified using monoavidin gel and subjected to trypsin digestion. The fragmented peptides were fractionated by nano-HPLC and identified using MALDI-TOF/TOF mass spectrometer.

Supplementary Table S3.

Accession number	Name	Days 0	3	7	14
P05784	Keratin, type I cytoskeletal 18		+	+	+
P11679	Keratin, type II cytoskeletal 8		+	+	+
P08113	Endoplasmin		+	+	
Q8VCM7	Fibrinogen γ chain		+	+	
P11276	Fibronectin		+	+	
P84244	Histone H3.3		+	+	
Q61425	Hydroxyacyl-coenzyme A dehydrogenase, mitochondrial		+	+	
Q9WUA2	Phenylalanyl-tRNA synthetase β chain		+	+	
Q9WVE8	Protein kinase C and casein kinase substrate in neurons protein 2		+	+	
Q8VDJ3	Vigilin		+	+	
Q60597	2-oxoglutarate dehydrogenase, mitochondrial		+		
P97351	40S ribosomal protein S3a		+		
Q571I9	Aldehyde dehydrogenase family 16 member A1		+		
P01027	Complement C3		+		
P01029	Complement C4-B		+		
Q8BMF4	Dihydrolipoyllysine-residue acetyltransferase component of pyruvate dehydrogenase complex, mitochondrial		+		
Q9D2G2	Dihydrolipoyllysine-residue succinyltransferase component of 2-oxoglutarate dehydrogenase complex, mitochondrial		+		
Q8BG05	Heterogeneous nuclear ribonucleoprotein A3		+		
Q9Z2X1	Heterogeneous nuclear ribonucleoprotein F		+		
P17095	High mobility group protein HMG-I/HMG-Y		+		
P43274	Histone H1.4		+		
Q8CGP7	Histone H2A type 1-K		+		
P08071	Lactotransferrin		+		
O08663	Methionine aminopeptidase 2		+		
Q5SX40	Myosin-1		+		
Q9D6A1	Myosin-Ih		+		
Q62446	Peptidyl-prolyl cis-trans isomerase FKBP3		+		
Q8C0C7	Phenylalanyl-tRNA synthetase alpha chain		+		

Q61656	Probable ATP-dependent RNA helicase DDX5	+		
Q99JB8	Protein kinase C and casein kinase II substrate protein 3	+		
P62320	Small nuclear ribonucleoprotein Sm D3	+		
Q9CW03	Structural maintenance of chromosomes protein 3	+		
P40630	Transcription factor A, mitochondrial	+		
Q9JJZ2	Tubulin α-8 chain	+		
P99024	Tubulin β-5 chain	+		
Q62376	U1 small nuclear ribonucleoprotein 70 kDa	+		
Q921H8	3-ketoacyl-CoA thiolase A, peroxisomal		+	
Q61147	Ceruloplasmin		+	
O35658	Complement component 1 Q subcomponent-binding protein, mitochondrial		+	
P01942	Hemoglobin subunit α		+	
Q8R1M2	Histone H2A.J		+	
Q8BG05-2	Isoform 2 of Heterogeneous nuclear ribonucleoprotein A3		+	
Q497I4	Keratin, type I cuticular Ha5		+	
P11247	Myeloperoxidase		+	
Q8VDD5	Myosin-9		+	
P62960	Nuclease-sensitive element-binding protein 1		+	
P35700	Peroxiredoxin-1		+	
P29341	Polyadenylate-binding protein 1		+	
Q810M5	Probable palmitoyltransferase ZDHHC19		+	
P27005	Protein S100-A8		+	
P31725	Protein S100-A9		+	
P21981	Protein-glutamine γ-glutamyltransferase 2		+	
Q91X83	S-adenosylmethionine synthase isoform type-1		+	
Q921I1	Serotransferrin		+	
Q9WUM5	Succinyl-CoA ligase [GDP-forming] subunit α, mitochondrial		+	
P68373	Tubulin α-1C chain		+	
Q62452	UDP-glucuronosyltransferase 1-9		+	
Q61646	Haptoglobin			+
Q8CGP6	Histone H2A type 1-H			+
Q6GSS7	Histone H2A type 2-A			+
Q8CGP1	Histone H2B type 1-K			+
Q02257	Junction plakoglobin			+
P17897	Lysozyme C-1			+

**P08905**	**Lysozyme C-2**	**+**
**P26041**	**Moesin**	**+**

Supplementary Table S3. Identified possible substrates for TGs using BPA

Liver extract on each indicated day after BDL surgery was incubated with BPA. The peptide-incorporated proteins were then purified using monoavidin gel and subjected to trypsin digestion. The fragmented peptides were fractionated by nano-HPLC and identified using MALDI-TOF/TOF mass spectrometer. The newly identified possible substrates in each indicated day were demonstrated as “+” compared to control sample (Day 0).

Supplementary Table S4.

Accession number	Name
Q9CQ62	2,4-dienoyl-CoA reductase, mitochondrial
O08756	3-hydroxyacyl-CoA dehydrogenase type-2
Q8BWT1	3-ketoacyl-CoA thiolase, mitochondrial
P20029	78 kDa glucose-regulated protein
Q8QZT1	Acetyl-CoA acetyltransferase, mitochondrial
P63260	Actin, cytoplasmic 2
P47738	Aldehyde dehydrogenase, mitochondrial
Q61176	Arginase-1
P16460	Argininosuccinate synthase
Q03265	ATP synthase subunit α, mitochondrial
P56480	ATP synthase subunit β, mitochondrial
O35490	Betaine--homocysteine S-methyltransferase 1
Q8C196	Carbamoyl-phosphate synthase [ammonia], mitochondrial
P24270	Catalase
Q9QZQ8	Core histone macro-H2A.1
Q99LB2	Dehydrogenase/reductase SDR family member 4
Q91Y97	Fructose-bisphosphate aldolase B
Q60759	Glutaryl-CoA dehydrogenase, mitochondrial
P16858	Glyceraldehyde-3-phosphate dehydrogenase
P63017	Heat shock cognate 71 kDa protein
O88569	Heterogeneous nuclear ribonucleoproteins A2/B1
P63158	High mobility group protein B1
Q8CGP2	Histone H2B type 1-P
P62806	Histone H4
Q61781	Keratin, type I cytoskeletal 14
Q6IFX2	Keratin, type I cytoskeletal 42
Q922U2	Keratin, type II cytoskeletal 5
Q922Q8	Leucine-rich repeat-containing protein 59
Q99J39	Malonyl-CoA decarboxylase, mitochondrial
P24369	Peptidyl-prolyl cis-trans isomerase B
Q9R0H0	Peroxisomal acyl-coenzyme A oxidase 1
Q9DBM2	Peroxisomal bifunctional enzyme
P51660	Peroxisomal multifunctional enzyme type 2
Q99MZ7	Peroxisomal trans-2-enoyl-CoA reductase

**Q01405**	**Protein transport protein Sec23A**
**P26043**	**Radixin**
**Q99PL5**	**Ribosome-binding protein 1**
**Q78PY7**	**Staphylococcal nuclease domain-containing protein 1**
**P38647**	**Stress-70 protein, mitochondrial**
**P80318**	**T-complex protein 1 subunit γ**
**Q9D0R2**	**Threonyl-tRNA synthetase, cytoplasmic**
**P50544**	**Very long-chain specific acyl-CoA dehydrogenase, mitochondrial**

Supplementary Table S4. Identified possible substrates for TGs in control sample

Liver extract on control sample (Day 0) was incubated with BPA. The peptide-incorporated proteins were then purified using monoavidin gel and subjected to trypsin digestion. The fragmented peptides were fractionated by nano-HPLC and identified using MALDI-TOF/TOF mass spectrometer.
